# The *Vibrio cholerae* Minor Pilin TcpB Initiates Assembly and Retraction of the Toxin-Coregulated Pilus

**DOI:** 10.1371/journal.ppat.1006109

**Published:** 2016-12-19

**Authors:** Dixon Ng, Tony Harn, Tuba Altindal, Subramania Kolappan, Jarrad M. Marles, Rajan Lala, Ingrid Spielman, Yang Gao, Caitlyn A. Hauke, Gabriela Kovacikova, Zia Verjee, Ronald K. Taylor, Nicolas Biais, Lisa Craig

**Affiliations:** 1 Department of Molecular Biology and Biochemistry, Simon Fraser University, Burnaby, British Columbia, Canada; 2 Department of Microbiology and Immunology, Geisel School of Medicine at Dartmouth, Hanover, New Hampshire, United States of America; 3 Biology Department, Brooklyn College, City University of New York, Brooklyn, New York, United States of America; 4 Graduate Center, City University of New York, Brooklyn, New York, United States of America; University of California Davis School of Medicine, UNITED STATES

## Abstract

Type IV pilus (T4P) systems are complex molecular machines that polymerize major pilin proteins into thin filaments displayed on bacterial surfaces. Pilus functions require rapid extension and depolymerization of the pilus, powered by the assembly and retraction ATPases, respectively. A set of low abundance minor pilins influences pilus dynamics by unknown mechanisms. The *Vibrio cholerae* toxin-coregulated pilus (TCP) is among the simplest of the T4P systems, having a single minor pilin TcpB and lacking a retraction ATPase. Here we show that TcpB, like its homolog CofB, initiates pilus assembly. TcpB co-localizes with the pili but at extremely low levels, equivalent to one subunit per pilus. We used a micropillars assay to demonstrate that TCP are retractile despite the absence of a retraction ATPase, and that retraction relies on TcpB, as a *V*. *cholerae tcpB* Glu5Val mutant is fully piliated but does not induce micropillars movements. This mutant is impaired in TCP-mediated autoagglutination and TcpF secretion, consistent with retraction being required for these functions. We propose that TcpB initiates pilus retraction by incorporating into the growing pilus in a Glu5-dependent manner, which stalls assembly and triggers processive disassembly. These results provide a framework for understanding filament dynamics in more complex T4P systems and the closely related Type II secretion system.

## Author Summary

Bacterial pathogens utilize a number of highly complex and sophisticated molecular systems to colonize their hosts and alter them, creating customized niches in which to reproduce. One such system is the Type IV pilus system, made up of dozens of proteins that form a macromolecular machine to polymerize small pilin proteins into long thin filaments that are displayed on the bacterial surface. These pili have a remarkable array of functions that rely on their ability to (i) adhere to many substrates, including host cell surfaces, pili from nearby bacteria, DNA and bacterial viruses (bacteriophage), and (ii) to depolymerize or *retract*, which pulls the bacteria along mucosal surfaces, pulls them close together in protective aggregates, and can even draw in substrates like DNA and bacteriophage for nutrition and genetic variation. For most Type IV pilus systems, retraction is an energy-driven process facilitated by a retraction ATPase. We show here that in the simplest of the Type IV pilus systems, the *Vibrio cholerae* toxin-coregulated pilus, a pilin-like protein initiates pilus retraction by what appears to be mechanical rather than enzymatic means. Our results provide a framework for understanding more complex Type IV pili and the related Type II secretion systems, which represent targets for novel highly specific antibiotics.

## Introduction

*Vibrio cholerae* is a Gram-negative bacterial pathogen that causes the human diarrheal disease cholera, which afflicts millions of people each year [1]. Cholera is marked by copious watery diarrhea that can lead to dehydration, shock, organ failure and death as little as 24 hours after infection [2]. The severe diarrhea is caused by cholera toxin, an ADP ribosylating enzyme that is transported from the cytoplasm across the inner membrane via the Sec machinery and from the periplasm across the outer membrane via the Type II secretion (T2S) system [1, 3–5]. Colonization of the small intestine by *V*. *cholerae* requires a second virulence factor, the toxin co-regulated pilus (TCP), which self-associates to induce bacterial aggregation and microcolony formation [6–8]. In addition to building a pilus filament, the TCP assembly apparatus acts like a T2S system, exporting a soluble protein, TcpF, across the outer membrane [9, 10]. TCP is also the primary receptor for the lysogenic bacteriophage CTXϕ, which carries the cholera toxin genes *ctxAB* and is responsible for converting *V*. *cholerae* from a harmless marine microbe to a deadly human pathogen causing pandemic disease [11]. Understanding TCP biology is essential for understanding *V*. *cholerae* pathogenesis and designing prevention and treatment strategies for cholera disease.

TCP are members of the Type IV pilus (T4P) class, which are expressed by many Gram-negative bacteria as well as some Gram-positive species and archaea [12–16]. T4P perform a myriad of functions including adhesion, microcolony formation, phage and DNA uptake, twitching motility and protein secretion. They are polymers comprised primarily of a single protein subunit, the major pilin. X-ray crystal structures of full length major pilins reveal a small protein with a curved ~53-residue α-helical spine, α1, embedded via its C-terminal half, α1C, in an antiparallel β-sheet within the globular C-terminal domain of the protein [14, 17–20]. The N-terminal half of α1, α1N, is an exposed mostly hydrophobic stalk with an invariant glutamate at position 5. Pilin subunits are arranged in the pilus filament in a helical array with the α1Ns associated in the hydrophobic core of the pilus and Glu5 positioned to neutralize the positively charged N-terminal amine (N1+) of an adjacent subunit [18, 21, 22]. Two T4P sub-classes have emerged, Type IVa (T4a) and Type IVb (T4b). The T4b pili are present on enteric bacteria such as *V*. *cholerae*, enterotoxigenic *Escherichia coli* (ETEC), enteropathogenic *E*. *coli* (EPEC) and *Salmonella* Typhi as well as the Flp pili and R64 thin pilus [23, 24]. The T4b pili are distinguished from the T4a pili of *Myxococcus xanthus*, *Pseudomonas aeruginosa* and the pathogenic Neisseria in having fewer assembly components, all of which are encoded on a single gene cluster, a longer signal peptide and mature region for the major pilin and a distinct connectivity for the β-sheet in the globular domain [14].

Polymerization of the major pilin subunits into surface-displayed pili is accomplished by an assembly machinery that spans the bacterial envelope. This machinery is comprised of as few as 10 proteins to 30 or more components [16]. Despite recent major structural advances [25, 26] our understanding of T4P assembly remains incomplete. Major pilins are synthesized as prepilins, which are translocated across the inner membrane and are simultaneously processed by a dedicated prepilin signal peptidase that removes the signal peptide, leaving the subunits anchored in the inner membrane via their hydrophobic α1N [27–30]. Pilin subunits are thought to dock into the base of a growing pilus, which allows α1 to transition seamlessly from the acyl phase of the inner membrane to the hydrophobic core of the pilus where it is surrounded by its neighboring α1s [18]. The docked pilin is prevented from diffusing away from the pilus by extrusion of the growing pilus a short distance of 8–10 Å out of the membrane, equivalent to the axial rise per subunit [18, 21, 22], which opens up a gap around the base of the filament for the next subunit to dock. Polymerization is powered by ATP hydrolysis by the assembly ATPase located on the cytoplasmic side of the inner membrane [31–34]. Efficient pilus assembly requires the highly conserved Glu5, as amino acid substitutions at this position substantially reduce or eliminate pilus assembly [22, 35–37]. We have proposed that an electrostatic attraction between Glu5 on the incoming pilin subunit and the positively-charged N-terminal amino group on the terminal subunit in the growing pilus (N1+) contributes to subunit docking by neutralizing these charges in the acyl phase of the lipid bilayer prior to their extrusion from the membrane as part of the pilus [18, 22]. A bridge of electron density is observed connecting the N-terminal α-helices in the recent ~ 6 Å reconstruction of the *Neisseria meningitidis* T4P cryo-electron microscopy reconstruction, supporting the existence of a Glu5:N1+ salt bridge in the assembled pilus [21].

In Gram-negative bacteria T4P filaments grow across the periplasm and through the outer membrane via a complex conduit comprised of an outer membrane secretin channel and a network of proteins that connect this channel to the inner membrane assembly platform [38–41]. In addition to the assembly ATPase, most T4P systems possess a second “retraction” ATPase, often called PilT, which is required to depolymerize or retract the pili by an unknown mechanism [33, 42–46]. Retraction allows twitching motility, which pulls the bacteria along surfaces, and can draw bound substances like DNA and bacteriophage into the cell [47–53]. Retraction has been demonstrated, both directly and indirectly for *P*. *aeruginosa*, *Neisseria gonorrhoeae*, *M*. *xanthus* and *Streptococcus sanguinis* T4P by total internal reflection fluorescence microscopy, optical tweezers and elastic micropillars assays [52, 54–61]. *N*. *gonorrhoeae* pili can retract at an astonishing rate of ~1 μm/sec, equivalent to removing ~1000 pilin subunits per second. *N*. *gonorrhoeae* and *M*. *xanthus* T4P retract with forces of 100–150 pN in a PilT-dependent manner and thus PilT is considered to be the strongest molecular motor known [58]. Pilus retraction likely occurs by reversing pilus assembly, whereby subunits translocate from the base of the growing pilus into the inner membrane.

The T4P system is structurally and functionally related to the T2S system, whereby major “pseudopilins” assemble into a pseudopilus that grows from the inner membrane through the periplasm, driven by an assembly ATPase [4, 62–64]. While T4P are long thin filaments displayed on the bacterial surface, T2S pseudopili normally do not grow across the outer membrane: they are thought to form periplasmic pilus stubs that are rapidly assembled and disassembled, producing a piston-like motion to extrude substrates through the secretin channel [64–68]. Pseudopili can, however, be induced to grow into long surface-displayed pili when the major pseudopilin or the entire T2S operon are overexpressed [69, 70]. The T2S major pilins resemble Type IV pilins, having a hydrophobic N-terminus with Glu5 and a canonical pilin fold [71]. Despite the model for a dynamic pseudopilus, T2S systems lack a retraction ATPase to facilitate depolymerization.

In addition to the major pilin, the T4P and T2S systems each possess several minor pilins, pilin-like proteins that share N-terminal sequence homology and are structurally similar to their corresponding major pilins but are much less abundant in the cell. Minor pilins are known to be involved in T4P and T2S dynamics and functions but a mechanistic understanding is still lacking. Four core minor pilins, GspH, GspI, GspJ and GspK, are common to both systems, though their names differ depending on the system and bacterial species. T4P GspHIJK orthologs in *P*. *aeruginosa*, *N*. *gonorrhoeae* and EPEC are required for pilus assembly in an otherwise wild type (WT) background [72–74], but some pili are produced in minor pilin mutants that also lack the retraction ATPase, PilT [75–78]. Thus, it has been proposed that the minor pilins function as effectors of pilus homeostasis in the presence of the retraction ATPase rather than as essential initiators of pilus assembly [76]. Some T4P systems also possess one or more non-core minor pilins that are dispensable for pilus assembly but are required for pilus functions, including PilE in *P*. *aeruginosa* [77], PilV in *N*. *gonorrhoeae* [79] and ComP, PilX and PilV *in N*. *meningitidis* [80–86]. The T4P minor pilins localize to the pilus fraction and are shown by immunogold labeling and transmission electron microscopy (immunogold TEM) in some cases to be incorporated into the pilus [74, 82]. Structural studies on these minor pilins reveal the canonical pilin fold, consistent with their ability to incorporate into the pilus filament [77, 80, 82, 87–89].

Like the minor pilins of the T4P system, the T2S system minor pilins function in initiation of filament assembly [68, 70, 90–93]. Glu5 is conserved in most T2S minor pilins and was shown for at least one of these, PulH of *Klebsiella oxytoca*, to be necessary for secretion [94]. ETEC GspI, GspJ and GspK were crystallized in a ternary complex that is proposed to assemble at the tip of the pseudopilus [4, 95], consistent with a role in initiating pseudopilus assembly. However this minor pilin tip complex may also prevent growth of the pseudopilus through the secretin channel. The largest of the ETEC minor pseudopilins, GspK, has a bulky α-helical domain inserted between the first two strands of the pilin domain β-sheet. This large protein at the tip of the pilus may provide a steric or chemical signal that stalls pseudopilus assembly upon contact with the secretin channel [95]. ETEC GspK is a member of the GspK family of pilin-like proteins that are larger than their corresponding minor pilins and lack a position 5 glutamate [96]. Consistent with the proposed role in pseudopilus growth arrest, the GspK minor pilin XcpX was shown to control pseudopilus length in a *P*. *aeruginosa* T2S mutant overexpressing the major pseudopilin XcpT: longer more abundant pseudopili were produced in this strain when *xcpX* was deleted whereas fewer pseudopili were produced when *xcpX* was overexpressed [97]. Blocking passage of the pseudopilus through the secretin channel may trigger disassembly, producing a piston-like motion that extrudes protein substrates across the secretin channel independent of a retraction ATPase.

The closely related *V*. *cholerae* TCP and the ETEC CFA/III T4b pili provide comparatively simple systems in which to study the role of the minor pilins in filament dynamics as their assembly machinery has fewer than a dozen components, with only a single minor pilin. The functions associated with these pili—secretion, aggregation and phage transduction—suggest dynamic filaments that rapidly assemble and retract, as seen for the more complex T2S and T4P systems, yet they, like the T2S systems, lack a retraction ATPase. We show here that *V*. *cholerae* TCP are indeed retractile and that retraction is induced by the single minor pilin, TcpB. We further show that TcpB, like its ETEC counterpart CofB [98, 99], induces pilus assembly. Efficient TCP retraction but not assembly requires the conserved position 5 glutamate, consistent with TcpB having both a tip-associated localization, like the GspK family pseudopilins, but also incorporating into the growing pilus in the place of a major pilin. Our findings in the T4b pilus system provide important insights for understanding pilus dynamics in the more complex T4P and T2S systems.

## Results

### *V*. *cholerae* TcpB is a pilin-like protein

**Fig 1:**
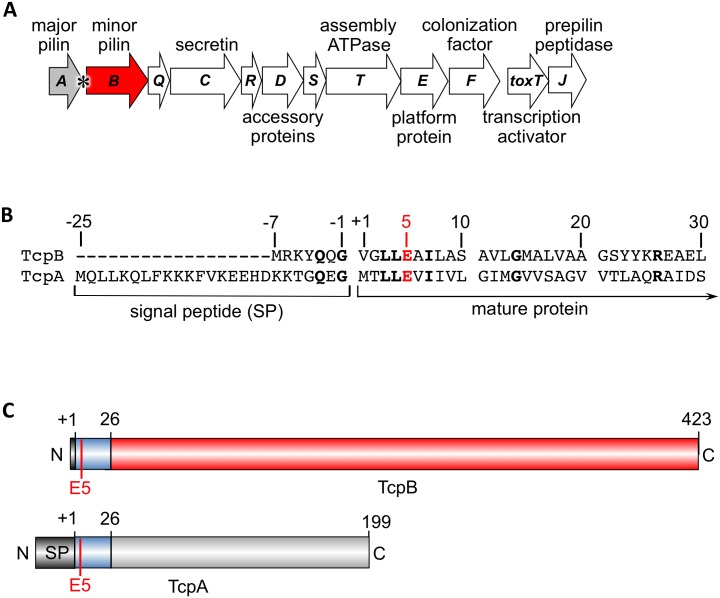
*V*. *cholerae tcp* operon and comparison of the minor pilin TcpB and the major pilin TcpA. (A) *tcp* operon with the major pilin gene *tcpA* colored grey and the minor pilin gene *tcpB* colored red. The putative rho-independent transcription terminator is indicated with an asterisk. **(B)** Amino acid sequence alignment between the signal peptide and the N-terminal 30 residues of TcpB and TcpA (NCBI accession, CAA45456 and CAA45455, respectively). **(C)** Schematic of TcpB and TcpA pre-proteins.

TcpB is encoded in the *tcp* operon immediately downstream of the *tcpA* gene encoding the major pilin subunit, TcpA (Fig 1A). TcpB is predicted to have a 7-amino acid signal peptide, which is considerably shorter than the 25-residue signal peptide of TcpA, and a 423 amino acid mature protein (46 kDa), which is much larger than the 199-residue TcpA (20 kDa, Fig 1B and 1C). The TcpB mature N-terminus shares amino acid sequence similarity with the N-terminal 25-residue polymerization domain of TcpA, including the conserved Glu5 (Fig 1B), but differs from TcpA in the C-terminal region. Importantly, *tcpA* has a putative rho-independent transcription termination site at its 3’ end (Fig 1A) [100], allowing it to be transcribed at higher levels than *tcpB* and other genes in the *tcp* operon. Apart from TcpA, TcpB is the only pilin-like protein encoded in this operon.

### TcpB is required for efficient pilus production

**Fig 2:**
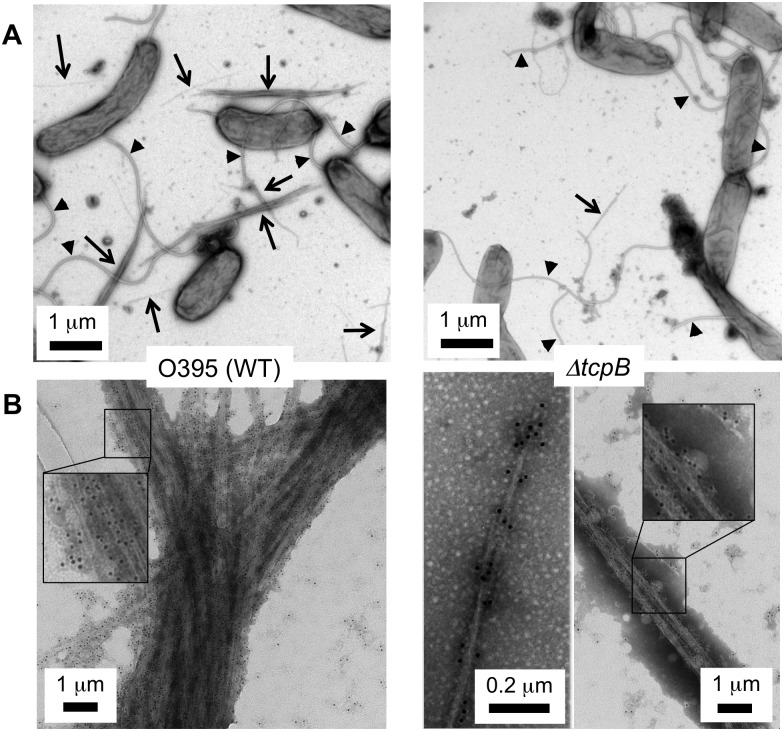
TCP are produced in very low numbers in the *V*. *cholerae* Δ*tcpB* strain. **(A)** The left TEM image is representative of WT O395, in which TCP bundles are abundant, indicated by arrows. In contrast, very few TCP bundles are observed for the Δ*tcpB* strain. One small bundle is shown in the image on the right. Flagella are indicated with arrowheads. **(B)** TEM images of TCP labeled with anti-TcpA primary antibody and gold-labeled secondary antibody. The gold particles are 6 nm in diameter. A section of each image is magnified in the inset to show the gold particles attached to the pili.

Previous reports indicate that TcpB is essential for TCP production [101–103]. We show here that the Δ*tcpB* strain can in fact assemble pili but at very low levels. Low magnification TEM images show abundant thick TCP bundles for *V*. *cholerae* WT strain O395, whereas few TCP are observed for the Δ*tcpB* mutant (Fig 2A). Nonetheless, individual TCP filaments are morphologically indistinguishable between the two strains. Pili from the WT and Δ*tcpB* strains were probed with polyclonal anti-TcpA primary antibodies and gold-labeled secondary antibodies and imaged by TEM. Though much less abundant, the Δ*tcpB* pili observed by TEM are gold labeled (Fig 2B), confirming that they are indeed TCP.

**Fig 3:**
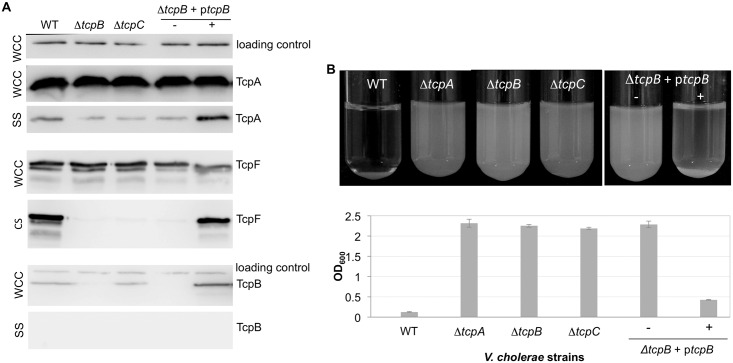
*V*. *cholerae* requires TcpB for efficient pilus assembly and functions. **(A)** Immunoblots of *V*. *cholerae* whole cell culture (WCC), sheared culture supernatant (SS) and cleared culture supernatant (CS) fractions probed with antibodies against TcpA, TcpF and TcpB, as indicated by the labeled protein bands. SS for the TcpA and TcpB blots was prepared by homogenizing the cells to shear the pili then removing intact cells by centrifugation. The SS fractions were topped up with PBS to a volume matching that of the WCC, and equal volumes (25 ul) of each sample were loaded onto the gel. TcpA in the SS fraction of the Δ*tcpB* and Δ*tcpC* mutants may represent contamination from cell membranes due to the shearing method or membrane blebbing due to high levels of TcpA that accumulate in the inner membrane. Secreted TcpF is detected in the CS fraction, obtained by removing *V*. *cholerae* cells by centrifugation and filtration without shearing. TcpF often appears as a doublet in immunoblots. The lower band of the doublet appears to be a proteolysed form of TcpF lacking the N-terminal ~25 amino acids. This segment is not resolved in the TcpF crystal structure suggesting it is flexible, which may explain its protease sensitivity [10]. The Δ*tcpB* strain is complemented with p*tcpB* without (-) and with (+) 0.001% rhamnose inducer. The loading control is an unknown ~60 kDa protein present in the WCC fraction and detected by the *Strep*-Tactin-HRP conjugate. **(B)** *V*. *cholerae* autoagglutination as a function of TcpB expression. Cell cultures are shown after overnight growth followed by 15 min stationary incubation at room temperature (top panel). The OD_600_ of the culture supernatant after the cells have settled is plotted for each sample in the bottom panel. The more complete the autoagglutination the lower the OD_600_ value. Values are averaged for 3 experiments; error bars represent standard deviations.

To evaluate whether TcpB influences pilus production by acting at the level of TcpA expression and/or stability, *V*. *cholerae* overnight cultures were examined by SDS-PAGE and immunoblotting. TcpA levels in whole cell culture samples (WCC), which represent the total protein within the cells and in the supernatant, are comparable in the WT O395 and Δ*tcpB* strains (Fig 3A), suggesting that TcpB is required for pilus assembly rather than for optimal expression and stability of TcpA. We also looked at the levels of TcpA in the culture supernatant after homogenizing the cells to shear off their pili (sheared cell supernatant, SS). Immunoblotting of the SS fraction with anti-TcpA antibodies showed low levels of TcpA for WT *V*. *cholerae* compared to the WCC fraction. This is likely due to the tendency of TCP to bundle, which makes them difficult to separate from the cells as the bundles pellet with the cells even at low *g*-forces. Nonetheless, TcpA levels were further reduced in the Δ*tcpB* mutant, comparable to that of a Δ*tcpC* mutant lacking a functional secretin channel. TcpA levels in the Δ*tcpB* SS fraction were restored when TcpB was expressed on a plasmid, p*tcpB*, from a rhamnose-inducible promoter P_RHA_ (Fig 3A). Taken together, the immunoblots and TEM results suggest that TcpB, like the closely related ETEC CofB and other minor pilins [76, 78, 92, 98, 104], is involved in initiating pilus assembly.

TCP-mediated functions of TcpF secretion and bacterial autoagglutination were analyzed for the Δ*tcpB* mutant and complemented strain. TcpF secretion is assayed by immunoblotting cleared culture supernatant (CS), in which overnight *V*. *cholerae* cultures are centrifuged and filtered to remove bacteria. TcpF secretion is abrogated in the Δ*tcpB* strain, comparable to that of the Δ*tcpC* strain, and is restored when TcpB is expressed ectopically (Fig 3A). Autoagglutination in overnight cultures can be assessed visually by examining the size and compactness of the macroscopic aggregates and the clarity of the supernatant, and more quantitatively by measuring the optical density (600 nm) of the culture supernatant, where the more complete the autoagglutination the lower the OD_600_ (Fig 3B). This assay serves as an in vitro readout for *V*. *cholerae* microcolony formation [8, 35]. Like TcpF secretion, autoagglutination is disrupted in the Δ*tcpB* strain and restored in the *tcpB*-complemented strain. The loss of pilus functions in the Δ*tcpB* mutant is consistent with a disruption of TCP assembly.

Because TcpB is homologous to TcpA in its N-terminal segment, and to ETEC CofB, which has a pilin domain [98], this minor pilin may incorporate into the pilus filament, most likely at its tip, consistent with its role in initiating pilus assembly. To test if TcpB co-localizes with TCP, WCC and SS fractions were immunoblotted with anti-TcpB antibody. As expected, TcpB is detected in the WCC fraction of WT and Δ*tcpC* strains and in the Δ*tcpB*+p*tcpB* strain, but not in the Δ*tcpB* strain (Fig 3A). While the intensities of the TcpA and TcpB bands cannot be directly compared as they are detected by different antibodies, the differences are nonetheless consistent with TcpB being produced in much lower levels than TcpA, as expected for a minor pilin. No TcpB was detected in the SS fraction for any of the strains (Fig 3A). However, based on the high ratio of TcpA:TcpB in the WCC fraction it is unlikely that TcpB would be detected in the SS fraction as so little TcpA is present in this fraction.

### TcpB is a minor component of the pilus

**Fig 4:**
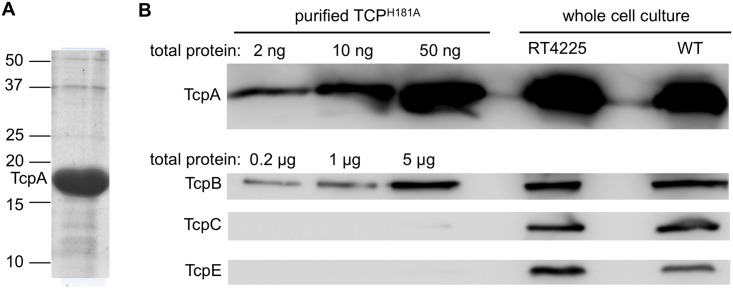
Co-localization of TcpB with purified TCP^H181A^. **(A)** Coomassie-stained SDS gel of TCP^H181A^ purified from *V*. *cholerae* RT4225, which has a mutation encoding a His181Ala substitution in the major pilin, TcpA. The protein concentration of the solution was found by spectrophotometry (A_280_) to be 10 mg/ml. The lane was overloaded with 90 μg of protein to show the purity, estimated at >95%. The positions of mass markers are indicated. The protein band at ~50 kDa is not likely to be TcpB (46 kDa), which based on the TcpA:TcpB stoichiometry determination (see below) is present in quantities too small to detect by Coomassie stain. **(B)** Immunoblots of pilins and pilus assembly components in purified TCP^H181A^ and whole cell culture. For the purified TCP^H181A^ samples, the total amount of protein loaded is indicated; for the whole cell culture samples, 25 μl was loaded into each well. Blots were probed with antibodies against TcpA and TcpB; TcpC and TcpE were also probed to assess contamination from outer and inner membranes, respectively. All blots were developed for 2 minutes.

Since the immunoblots for TcpA and TcpB from sheared supernatant fractions were inconclusive regarding co-localization of TcpB with the pili, we looked for TcpB in a more concentrated pilus sample. The *V*. *cholerae* mutant, RT4225, has a His181Ala substitution in the major pilin TcpA [35]. TCP^H181A^ pili can readily be purified by ammonium sulfate precipitation of the overnight culture supernatant [8, 22, 105]. This is likely because the pili don’t bundle and thus do not precipitate with the cells, and because they grow unusually long and fall off the bacteria into the culture supernatant during overnight growth. We can load much larger quantities of purified TCP^H181A^ onto a gel (Fig 4A) compared to our sheared supernatant fractions from WT *V*. *cholerae*, which increases our chance of detecting the low abundance TcpB. Purified TCP^H181A^ were examined by SDS-PAGE and immunoblotting with anti-TcpA and TcpB antibodies. TcpB was detected in the TCP^H181A^ pilus preparation when loaded in much larger amounts than that required to visualize the major pilin TcpA (Fig 4B), indicating that it is indeed a minor component of the pilus. *V*. *cholerae* RT4225 produces TcpA and TcpB in levels comparable to that of WT *V*. *cholerae* O395, as shown by immunoblots of whole cell culture (Fig 4B). To ensure that the TcpB present in the TCP^H181A^ preparation is not a contaminant of the *V*. *cholerae* envelope, this fraction was probed with antibodies against membrane components of the TCP assembly machinery: the outer membrane protein TcpC and the inner membrane protein TcpE. These proteins are almost undetectable even at the highest TCP^H181A^ concentration loaded. These results indicate that TcpB co-localizes with the pilus but is present in much lower levels than TcpA, consistent with it being a minor pilin.

To determine the TcpA:TcpB stoichiometry in the TCP^H181A^ fraction, each protein was quantified by comparing the intensities of the immunoblot bands with those of purified recombinant N-terminally truncated (ΔN-) TcpA and TcpB of known concentrations using densitometry. Band densities of known amounts of ΔN-TcpA and ΔN-TcpB were normalized and their averages were plotted; these plots were used to determine the amount of TcpA and TcpB in the TCP^H181A^ sample based on their densities (S1A and S1B Fig). The TCP^H181A^ sample was found by densitometry to contain 12±1 mg/ml of TcpA and 4±1 μg/ml of TcpB, giving a mass ratio of 3000:1 and a molar ratio of ~ 7000:1 TcpA:TcpB. WT TCP bundles are typically ~ 2–3 μm in length, whereas individual TCP^H181A^ pili appear to be longer but cannot be measured precisely as they extend past the edges of TEM images at magnifications at which they can be resolved (S1C Fig). Based on TCP having an axial rise of 8.4 Å [22], a ~ 6 μm-long pilus would be comprised of ~ 7000 TcpA subunits and only a single TcpB molecule. Since TcpB is involved in initiating pilus assembly we reason that this single minor pilin is located at the pilus tip. Interestingly, despite the very small amount of TcpB in the purified pilus fraction, TcpB is readily detected in whole cell culture by immunoblots (Figs 3A and 4B), indicating that more TcpB is made than is incorporated into the pili.

### *V*. *cholerae* TCP are retractile

**Fig 5:**
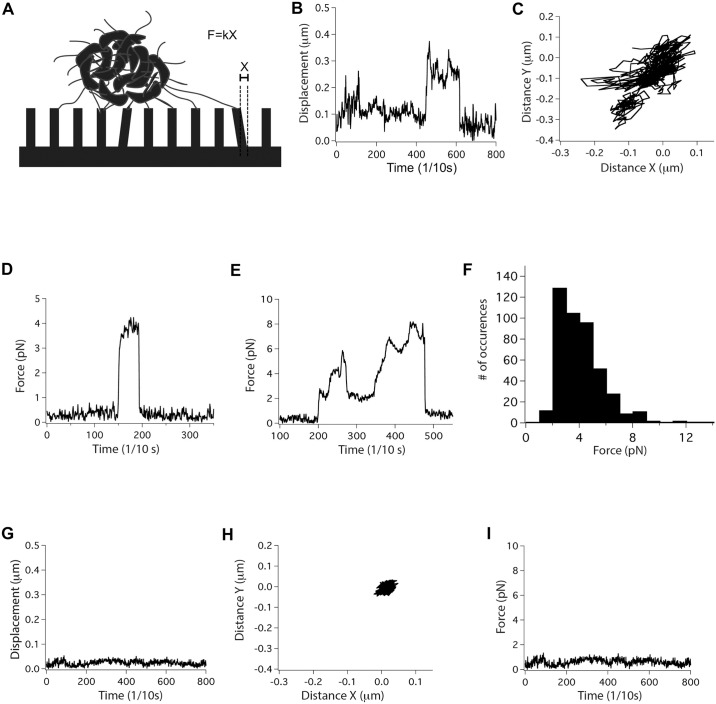
Micropillars assay demonstrates that TCP are retractile. **(A)** Illustration of the micropillars assay set-up. The force of retraction, *F*, is the product of the spring constant of the micropillars, *k* (here *k* = 25 pN/μm), and the displacement of a micropillar from its resting position, *X*. **(B-F)** Data plots from micropillars assays for *V*. *cholerae* Δ*flaA*. **(B)** Displacement of a single micropillar under a bacterial aggregate over time, measured from its resting position. The displacement is representative of hundreds of micropillars motions analyzed for several hours of recordings. **(C)** Projection in X and Y of the movement of the micropillars shown in (B) over the same period of time. **(D, E)** Representative values for force exerted on a single micropillar as a function of time for two separate retraction events. **(F)** Histogram of the force distribution for recorded TCP retraction events for *V*. *cholerae* Δ*flaA*. **(G-I)** Representative results for *V*. *cholerae* Δ*tcpB*/Δ*flaA* showing **(G)** the displacement of a single micropillar over time, which is not detected above noise level, **(H)** the projection in X and Y of the movement of the micropillars shown in (G), and **(I)** the calculated force exerted on the same micropillar over the same period of time.

We proposed previously that TCP might be retractile despite lacking a retraction ATPase, based on their functions in TcpF secretion, autoagglutination and phage uptake [106]. We wondered if TcpB might be involved in this process in addition to its role in initiating pilus assembly. Our hypothesis is that TcpB induces pilus retraction by randomly incorporating into a growing pilus in place of TcpA and stalling assembly, causing the pilus to spontaneously disassemble. Stalling could occur by TcpB blocking addition of subsequent major pilins into the growing pilus or by preventing passage of the pilus through the secretin channel, as was proposed for the ETEC minor pseudopilin GspK [95]. To test if TCP are indeed retractile we performed an elastic micropillars assay, which has been used to demonstrate and quantify retraction events for T4P of *N*. *gonorrhoeae* [54, 107] and *Streptococcus sanguinis* [57]. Bacterial microcolonies are placed on an array of elastic micropillars and the movements of the tips of the micropillars under and adjacent to a microcolony are observed by differential interference contrast microscopy (Fig 5A). To assess TCP retraction in *V*. *cholerae* we employed a mutant lacking the flagellin gene, Δ*flaA*, to eliminate non-pilus-mediated micropillar movement that might result from flagellar rotation. Movements of micropillars directly under and within a few microns of microcolonies were observed in the direction of the microcolonies, consistent with TCP binding and retraction (Fig 5B and 5C and S1 and S2 Movies). The force exerted on the micropillars, *F*, is the product of the calibrated elastic spring constant of the micropillars, *k*, and the displacement, *X*, of micropillars from their resting position (Fig 5A, 5D–5F). Although many micropillars motions were recorded, only those movements whereby the micropillars returned to their resting position were included in the force calculations. Micropillars motions occur in single pulls lasting a few seconds (Fig 5D) and also in more complex pulls that last tens of seconds (Fig 5E). Such complex patterns showing different local force maxima and plateaus could be explained by the pulling of multiple pili, perhaps within TCP bundles, as seen for *N*. *gonorrhoeae* T4P [54]. This study does not distinguish between movements induced by individual pili and pilus bundles. The average force for recorded pulling events is 4 ± 1.7 pN (n = 447, Fig 5F), which is substantially weaker than the 50–100 pN forces observed for individual T4P from *N*. *gonorrhoeae* and *S*. *sanguinis* [54, 57, 58, 107].

Next, we examined the *V*. *cholerae* Δ*tcpB*/Δ*flaA* strain, which produces very few pili, to demonstrate that the micropillars movements observed for *V*. *cholerae* Δ*flaA* are pilus-mediated. Since this strain does not aggregate, these bacteria were treated with anti-lipopolysaccharide antibody prior to the micropillars assay to promote cell-cell aggregation and microcolony formation to better mimic the *V*. *cholerae* Δ*flaA* conditions. No micropillar movements were observed under these artificially induced *V*. *cholerae* Δ*tcpB*/Δ*flaA* microcolonies (Fig 5G–5I and S3 Movie). These results confirm that the micropillars movements observed for the *V*. *cholerae* Δ*flaA* strain are pilus-dependent, supporting our hypothesis that TCP are retractile despite lacking a retraction ATPase.

### TcpB Glu5 is not required for pilus assembly but is required for pilus-mediated functions

**Fig 6:**
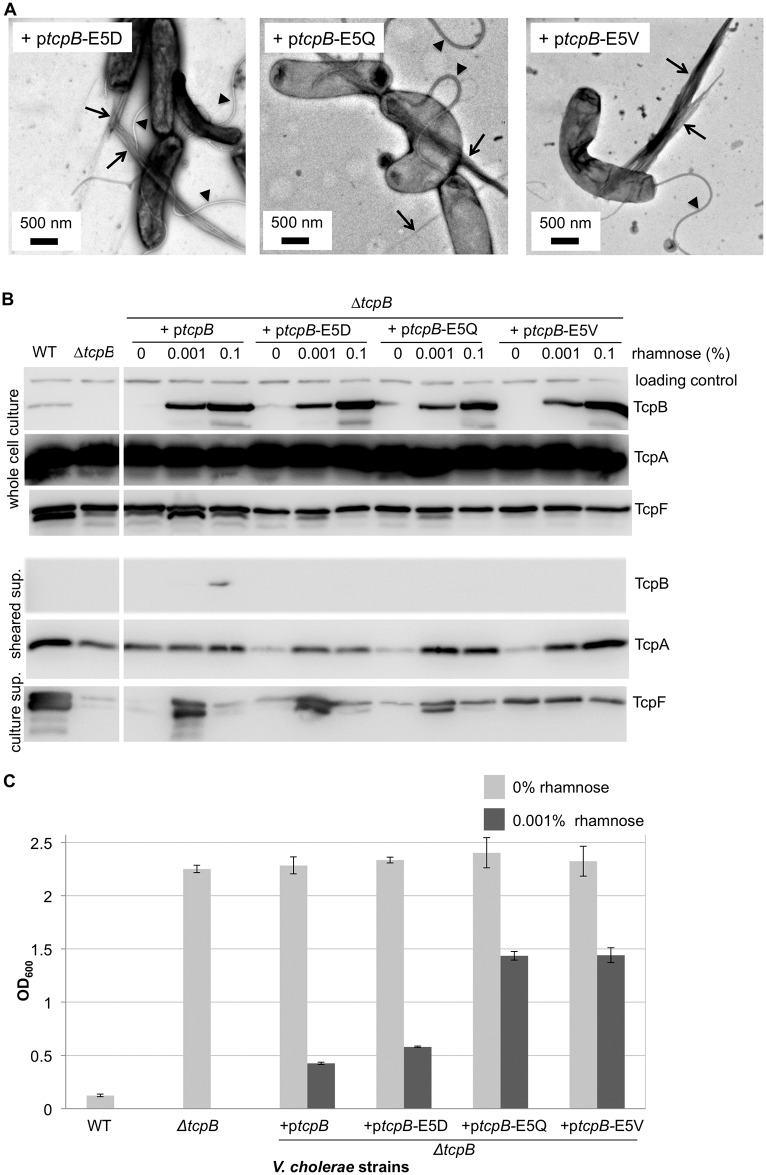
Complementation of *V*. *cholerae* Δ*tcpB* with *tcpB*-E5V mutants results in impaired autoagglutination and TcpF secretion without disrupting pilus assembly. **(A)** TEM images of *V*. *cholerae* Δ*tcpB* complemented with *tcpB* mutants encoding Glu5 substitutions show abundant pilus production. Arrows point to TCP bundles and arrowheads point to flagella. **(B)** Immunoblots of *V*. *cholerae* fractions for the Δ*tcpB* strain complemented with WT *tcpB* or *tcpB* mutants. TcpF secretion is disrupted for the TcpB Glu5 variants despite them producing TcpB levels comparable to that of the WT *tcpB*-complemented Δ*tcpB* strain. **(C)** Autoagglutination is impaired in the *V*. *cholerae* Δ*tcpB* strain rescued with TcpB Glu5 mutants. The lower the OD_600_ values the more complete the autoagglutination. Values are averaged for three replicates; error bars represent standard deviations.

To determine whether or not TcpB influences pilus retraction it was necessary to identify a *V*. *cholerae tcpB* mutant capable of assembling TCP at WT levels, but not retracting them. We showed previously that the conserved Glu5 of major pilin TcpA is necessary for efficient TCP assembly [22]. We reasoned that TcpB should not require Glu5 to initiate pilus assembly as it would be the first subunit in the pilus, but Glu5 would be necessary for TcpB to incorporate into the growing pilus to initiate retraction. Retraction-deficient TCP would be unable to pull bacteria into compact microcolonies, to produce a piston-like motion to secrete TcpF, or to draw CTXϕ phage into the bacterium. To test the role of TcpB Glu5 in pilus assembly, retraction and functions we generated a series of *tcpB* mutants with position 5 substitutions and examined their ability to rescue pilus assembly and functions in a Δ*tcpB* mutant. Expression of the TcpB Glu5 variants in a Δ*tcpB* mutant restored pilus assembly: these strains produce WT or better levels of TCP, as assessed by TEM (Fig 6A). However, pilus-mediated TcpF secretion and autoagglutination are impaired in the complemented strains (Fig 6B and 6C). The degree of functional impairment depends on the nature of the Glu5 substitution: the Δ*tcpB* mutant complemented with the most conserved Glu5 substitution to the negatively charged aspartate (E5D) showed a slight reduction in both TcpF secretion and autoagglutination compared to the WT strain; the Glu5Gln (E5Q) variant, which results in loss of the negative charge but retains polarity, showed poor TcpF secretion and autoagglutination; and the Glu5Val (E5V) variant, which has a hydrophobic side chain at position 5, was substantially impaired for TcpF secretion and autoagglutination. These differences cannot be attributed to defective TcpB expression for the Glu5 variants, as TcpB-E5X levels are comparable to the WT rescue and in all cases are higher than for endogenous expression in the WT O395 strain (Fig 6B). Gao et al. showed that the *V*. *cholerae* chromosomal Glu5Val mutant is deficient in autoagglutination, TcpF secretion and CTXϕ transduction [102]. Thus, TcpB Glu5 is not required for pilus assembly but is required for optimal pilus functions.

**Fig 7:**
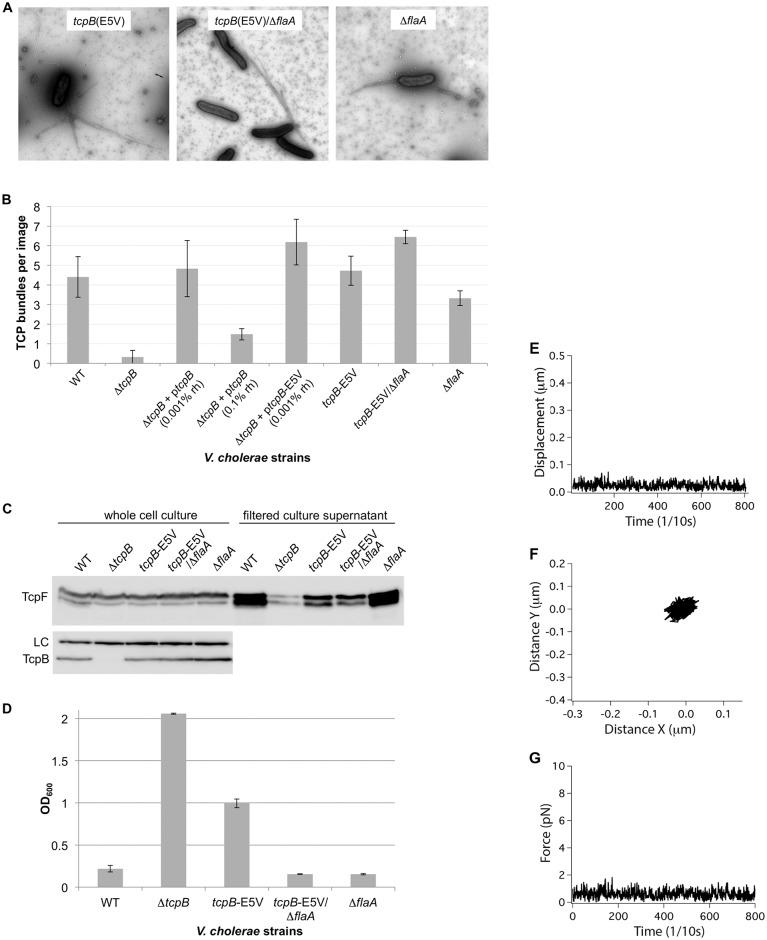
Autoagglutination, TcpF secretion and retraction but not TCP assembly are impaired in a chromosomal *tcpB*-E5V *V*. *cholerae* mutant. **(A)** TEM images of piliated *V*. *cholerae* chromosomal *tcpB*-E5V/*flaA* mutants. **(B)** TCP bundles in TEM images were counted to quantify pilus expression in the various *V*. *cholerae* strains. Sixteen or more low magnification (1850X) images were counted for three growth replicates for each strain; error bars represent standard deviations. **(C)** Immunoblots of TcpF and TcpB in whole cell culture and cleared culture supernatant for *tcpB*-E5V/*flaA* mutants. The Glu5Val substitution disrupts TcpF secretion without affecting TcpB production. **(D)** The Glu5Val substitution in TcpB disrupts autoagglutination, which is restored in the *tcpB*-E5V/*flaA* double mutant. The lower the OD_600_ values the more complete the autoagglutination. Values are averaged for three replicates; error bars represent standard deviations. **(E-G)** Micropillars assay data plots for *V*. *cholerae tcpB*-E5V/Δ*flaA* showing **(E)** the displacement of a representative micropillar as a function of time, which is not detected above noise level, **(F)** the projection in X and Y of the movement of this same micropillar over time, and **(G)** the force exerted on this micropillar over the same time period.

Interestingly, while low levels of TcpB expression (induction with 0.001% rhamnose) restored TcpF secretion to varying degrees depending on the nature of the Glu5 substitution, high levels (0.1% rhamnose) were detrimental for both the WT and the mutant *tcpB* complementation (Fig 6B). Induction with 0.001% rhamnose apparently provides the optimal level of TcpB expression for TCP functions. TCP bundles were quantified for both expression levels, revealing a substantial reduction in piliation for cells induced with 0.1% rhamnose, compared to those induced with 0.001% rhamnose (see Fig 7B). Thus, overexpression of TcpB disrupts pilus assembly, which impacts pilus functions. Even lower levels of rhamnose were tested for the complementation of the Δ*tcpB* strain with WT p*tcpB*, but these did not result in improved autoagglutination levels (S2 Fig). Together these results imply that a precise TcpA:TcpB stoichiometry must be attained for optimal pilus assembly and functions.

### Efficient TCP retraction requires TcpB Glu5

We hypothesized that the TcpB Glu5 variants are defective in inducing TCP retraction, explaining their impaired autoagglutination and TcpF secretion. To test this hypothesis we performed the micropillars assay on the *V*. *cholerae tcpB*-E5V mutant, which has the most severe functional defects. For these experiments we introduced the mutation encoding the Glu5Val substitution into the *tcpB* gene within the *V*. *cholerae* large chromosome via allelic exchange. Consistent with our results with ectopically expressed TcpB-E5V in the Δ*tcpB* strain (Fig 6A–6C), the chromosomal *tcpB*-E5V mutant produces pili that match WT TCP in morphology, bundling and quantity, as assessed by TEM (Fig 7A and 7B), but is impaired for both TcpF secretion and autoagglutination (Fig 7C and 7D). Interestingly, further deletion of *flaA* in the *tcpB*-E5V strain to eliminate flagella and non-pilus-mediated motility restored autoagglutination, though TcpF secretion remained impaired. Autoagglutination was also restored in another double mutant, *tcpB*-E5V/Δ*motAB*, which is non-motile but does produce flagella (S3 Fig). Micropillars pulling events were very infrequent for the *tcpB*-E5V/Δ*flaA* mutant (Fig 7E–7G and S4 Movie) despite it being piliated at WT levels (Fig 7A). Thus, the single amino acid substitution in TcpB, Glu5Val, appears to disrupt TCP retraction without disrupting TcpB-mediated pilus assembly. We propose that *V*. *cholerae* autoagglutination and microcolony formation are driven by dynamic cycles of elongation and retraction of TCP. Initially, weak complementary interactions among extended pili bring the bacteria in contact with one another [8], followed by pilus retraction to form tight aggregates. Without retraction the forces of flagellar rotation will disrupt the pilus:pilus interactions and prevent aggregation. Conversely, without flagellar rotation, weak pilus:pilus interactions may persist long enough for inefficient TcpB-E5V-mediated retraction or spontaneous non-TcpB-mediated retraction to occur, allowing the *tcpB*-E5V/Δ*flaA* mutant to autoagglutinate during overnight growth.

## Discussion

Our results represent an important conceptual advance in understanding pilus assembly and retraction and the role of the minor pilins in pilus dynamics for both the T4P and T2S pseudopilus systems. First, we show that the single minor pilin of the *V*. *cholerae* TCP system, TcpB, is required for efficient pilus assembly. Second, TCP are retractile despite lacking a retraction ATPase. Third, TCP retraction is induced by the minor pilin TcpB. Fourth, TcpB-mediated pilus retraction but not assembly requires TcpB-Glu5. Finally, retraction facilitates TCP-mediated processes of autoagglutination and TcpF secretion.

**Fig 8:**
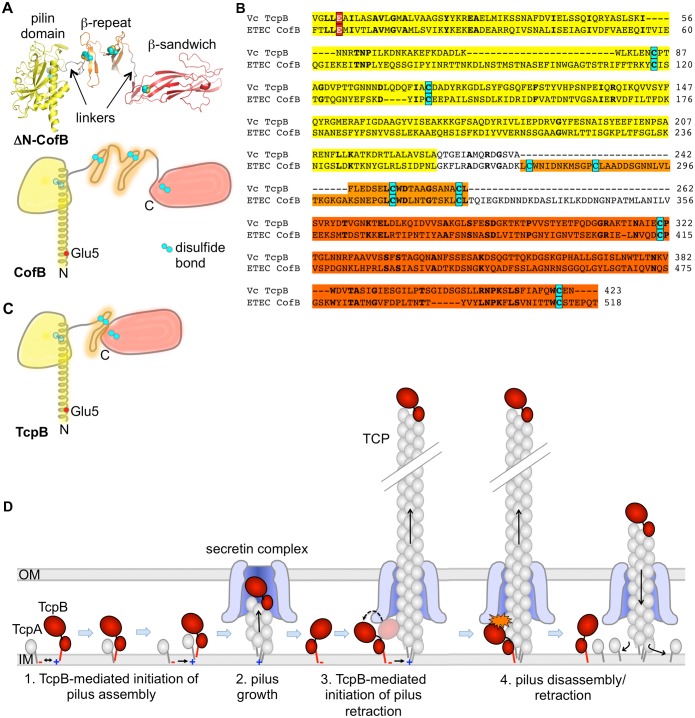
Comparison of TcpB and the ETEC minor pilin CofB and model for TcpB-mediated assembly and retraction. **(A)** Crystal structure of N-terminally truncated CofB (4QS4 [98]) shown in cartoon representation (top), and as a schematic (bottom) with the full N-terminal α-helix. Cysteines are colored cyan. **(B)** Amino acid sequence alignment of TcpB and CofB (NCBI Accession BAB62898). Alignment was first performed using Clustal Omega [110] then adjusted to align the cysteines. Identical residues are shown in boldface type. The conserved Glu5 (red with white text) and cysteines (cyan) are indicated. Discrete domains are shaded based on the coloring of CofB shown in (A). **(C)** Proposed schematic of the TcpB structure based on sequence alignment with CofB. **(D)** Model for TcpB-mediated initiation of pilus assembly and retraction. TcpB is represented as a pilin domain (red stick and small oval) with an additional C-terminal domain (large oval). Incorporation of TcpB into the growing pilus may block passage of the pilus through the secretin complex as shown (Steps 3, 4) or may prevent further incorporation of TcpA. If pilus assembly cannot proceed, the pilin subunits will melt back into the membrane, one subunit at a time, retracting the pilus.

The role of the single minor pilin in initiating pilus assembly was shown previously in the CFA/III and Longus T4b pilus systems of ETEC [98, 99, 108], which are closely related to *V*. *cholerae* TCP in sequence, structure, gene synteny and pilus functions [98, 109]. We proposed that the ETEC minor pilin CofB forms the first subunit in the growing pilus and is thus tip-associated [98]. CofB has a well-defined pilin domain that resembles its corresponding major pilin, CofA, in fold and size, but has an extended C-terminal region comprised of a β-repeat domain followed by a β-sandwich domain, which are connected by flexible linkers (Fig 8A) [98, 99]. Each domain is stabilized by one or more disulfide bonds. While sequence similarity between CofB and TcpB is low, alignment of the conserved N-terminal α1s and 6 cysteines reveals additional shared residues (Fig 8B) and suggests a similar structure for TcpB, but lacking the first β-repeat sub-domain and having only a single linker (Fig 8C). The tip of the pilus is the only location that this bulky pilin could occupy without undergoing a conformational change and without having the C-terminus protrude from the pilus. As tip-associated pilins TcpB and CofB could recruit major pilins and/or trigger opening of the outer membrane secretin channel to allow passage of the pilus to the cell surface (Fig 8D). While we have not directly demonstrated that TcpB is tip-associated, we show here that it co-localizes with purified TCP with approximately one molecule per pilus, consistent with this minor pilin incorporating into the pilus tip to initiate pilus assembly rather than acting at the level of the trans-envelope pilus biogenesis machinery. It is important to note that TcpB is not essential for pilus assembly as small amounts of pili are observed in the Δ*tcpB* mutant. It may be that the major pilin can itself nucleate TCP assembly and/or trigger opening of the secretin channel but does so inefficiently.

We show here that the non-motile but piliated *V*. *cholerae* strain Δ*flaA* induces micropillars movements, whereas the non-piliated Δ*tcpB*/Δ*flaA* strain does not, implying that micropillars movements are pilus-mediated. T4P-mediated micropillars movements have been demonstrated for *N*. *gonorrhoeae* and *S*. *sanguinis* [54, 57, 111], and T4P retraction has been demonstrated by optical tweezers experiments for *N*. *gonorrhoeae* and *M*. *xanthus* [52, 54, 56, 58, 59, 61]. Piliation is not sufficient for micropillars pulling as strain *tcpB*-E5V/Δ*flaA* makes better than WT levels of pili but micropillars movements were rarely observed for this strain, demonstrating a role for Glu5 in TCP retraction but not assembly. An alternative explanation for the lack of micropillars movements in *tcpB*-E5V/Δ*flaA*, that the Glu5Val substitution destabilizes the pili causing them to break off the cells, was ruled out based on the following observations: (i) TCP^TcpB-E5V^ bundles are more likely than WT TCP to be found by TEM attached to cells; (ii) TCP^TcpB-E5V^ levels in the overnight culture supernatant are approximately the same as TCP^WT^; and (iii) TCP^TcpB-E5V^ bundles tend to be longer than those of TCP^WT^, consistent with efficient pilus growth but inefficient retraction.

Our results with the *tcpB*-E5V strain, together with those of Gao et al. [102] show that pilus production alone is insufficient to mediate TCP functions of autoagglutination, TcpF secretion and CTXϕ uptake. Impaired functions in the *tcpB*-E5V strain correlate with lack of retraction, suggesting that TCP retraction is central to all of these processes: to produce the very tight, spherical aggregates observed for autoagglutinated *V*. *cholerae* in vitro and *V*. *cholerae* microcolonies in the intestines [35, 112]; to produce a piston-like motion to efficiently secrete TcpF; and to draw the filamentous CTXϕ into the periplasm. While the non-piliated Δ*tcpB* mutant is completely deficient in autoagglutination, TcpF secretion and phage uptake, the piliated *tcpB*-E5V mutant is able to perform these functions, though at substantially reduced levels, suggesting that pilus retraction occurs in this strain, perhaps inefficiently in a TcpB-E5V dependent manner, or by spontaneous TcpB-independent retraction. Nonetheless, it was surprising that the non-motile *tcpB*-E5V/Δ*flaA* mutant autoagglutinates as well as the WT strain. This observation highlights the disruptive force of flagellar rotation, which works against autoagglutination and microcolony formation. However TcpF secretion requires efficient retraction whether the cells are motile or not. It stands to reason that a pilus undergoing rapid cycles of elongation and retraction would extrude substrate more efficiently, whereas a pilus that grows long and then retracts would be more effective in establishing bacterial aggregates. TCP retract with forces averaging ~4 pN, enabling *V*. *cholerae* to move against moderate hydrodynamic flows to form compact aggregates and to draw phage into the periplasm. These forces are considerably weaker than the >100 pN forces exerted by *N*. *gonorrhoeae* and *M*. *xanthus* T4P [54, 56, 58], which perform twitching motility in addition to aggregation and phage uptake. The *N*. *gonorrhoeae* and *M*. *xanthus* T4P systems possess a retraction ATPase, which may influence the force, rate and frequency of the retraction events.

How might this minor pilin perform dual antagonistic roles in pilus biogenesis? We propose that TcpB incorporates at the pilus tip to initiate pilus assembly and remains at the pilus tip as the filament grows. Additionally TcpB incorporates transiently into the growing pilus where it causes assembly to stall, triggering disassembly or retraction. TcpB anchored in the inner membrane would occasionally insert into the growing pilus via its pilin domain, in place of TcpA (Fig 8D). The flexible linker would allow the bulky C-terminal domain to move out of the way of the pilin domain, forcing it onto the pilus surface where it would interfere with passage of the pilus through the secretin complex or perhaps prevent addition of the next major pilin, effectively stalling pilus assembly. Stalling would prevent incremental extrusion of the pilus so TcpB would eventually diffuse back into the inner membrane. As a result, the pilus would collapse a short distance into the membrane, whereupon the next pilin subunit would diffuse away, etc., disassembling the pilus one subunit at a time. The frequency of retraction events would depend on the molar ratio of TcpA to TcpB. This ratio would define the average length of the pilus: the more TcpB present the more frequent the retraction events and the fewer and shorter the pili. Our model infers that pilus retraction occurs as a consequence of the dynamic instability of the growing pilus filament, which requires energy from ATP hydrolysis to assemble but disassembles spontaneously, melting into the inner membrane one subunit at a time when assembly is interrupted, in this case by TcpB. This processive melting mechanism has been proposed by others [53], but was ruled out for T4P systems possessing a retraction ATPase as retraction visibly slows when ATP is depleted, suggesting an active role for the retraction ATPase [58]. While the mechanism of PilT-mediated retraction is not well understood, it clearly differs markedly from TcpB-mediated retraction, which, rather than utilizing chemical energy from ATP hydrolysis, appears to be a passive mechanical process that exploits the potential energy stored in the pilus.

Such a model explains why Glu5 is necessary for TcpB to induce pilus retraction but not pilus assembly. Docking of pilin subunits into a growing pilus is driven in part by charge complementarity between the N-terminal amine on the terminal subunit in the pilus and Glu5 on the incoming pilin (Fig 8D). As the first pilin subunit in the pilus, TcpB would need a positively charged N-terminus to neutralize the negatively charged Glu5 on an incoming TcpA, but would not itself need Glu5. However, to incorporate into the growing pilus TcpB would need Glu5 to neutralize N+ on the terminal TcpA at the base of the filament. Because TcpB-E5V can incorporate at the pilus tip it can initiate pilus assembly, thus the *tcpB*-E5V mutant is piliated, but TcpB-E5V cannot efficiently incorporate into the growing pilus to induce retraction, explaining the lack of micropillars motions and aberrant functions for this strain.

The sequence and functional similarities between *V*. *cholerae* TcpB and ETEC CofB suggest that the highly homologous ETEC minor pilins CofB and LngB also induce retraction of their respective T4b pili, CFA/III and Longus, respectively, to mediate aggregation and secretion. It is remarkable that a single minor pilin accomplishes both initiation and retraction, whereas four or more minor pilins work together to prime and in some cases to arrest pilus assembly in the more complex T4a pilus and T2S systems [74, 76, 77, 92, 93]. The extended C-terminal regions of the *V*. *cholerae* and ETEC minor pilins likely impart specialized functions accomplished by multiple minor pilins in the more complex systems, such as recruiting major pilins to initiate pilus assembly and/or opening the secretin channel. CofB, and by inference, TcpB, have flexible structures plus Glu5 that allow them to (i) incorporate at the tip of the pilus to initiate assembly, (ii) to pass through the secretin complex when tip-associated, and (iii) to incorporate into the growing pilus to trigger retraction. In contrast, the T2S minor pilin GspK is bulky but rigid and has a hydrophobic amino acid at position 5. While GspK in complex with GspHIJ appears to initiate pseudopilus assembly, its rigid structure might prevent its passage through the secretin complex [95], preventing growth of this pilus across the outer membrane, and triggering retraction. Rapid cycles of polymerization and retraction would serve to efficiently extrude substrates across the outer membrane. Importantly, its bulk, rigidity and lack of Glu5 would prevent GspK from incorporating at any site other than at the tip of the pilus. GspK is the only minor pilin of the GspHIJK cluster that lacks a glutamate at position 5. In fact, for most T2S and T4a pilus systems, as well as the EPEC T4b system of bundle-forming pili, only one of the four or five minor pilins has hydrophobic residue at position 5, the others having Glu5. In the T2S systems and the Gram positive T4P systems this minor pilin is also substantially larger than its corresponding major pilin, typical of GspK family pilins, and is encoded at the 3’ end of the minor pilin gene cluster. These features may represent signatures to identify minor pilins that form the first subunit in a nascent filament. Interestingly, for two of the minor pilins of the *K*. *oxytoca* T2S system, Glu5 has been implicated in interactions with inner membrane components of the pseudopilus assembly machinery, demonstrating a critical and complex role for this residue in filament formation [94].

The minimalist *V*. *cholerae* and ETEC T4b systems may represent precursors for other T4P and T2S systems. In these systems all genes necessary for T4b pilus assembly are clustered on a single operon. The *V*. *cholerae tcp* operon is present on a mobile genetic element, the Vibrio pathogenicity island [7], and the ETEC *cof* operon and *lng* operons are present on large virulence plasmids [113, 114]. These bacteria could pass their pilus operons to other bacteria during gut infections. Additional minor pilin genes likely arose via gene duplication. The T2S system appears more closely related to the *V*. *cholerae* and ETEC T4b pilus systems as T2S genes are also located on a single operon, they lack a retraction ATPase and they encode a large GspK family subunit. *V*. *cholerae* TcpB and ETEC CofB are large like the GspK family pilins but they have a flexible C-terminal region and Glu5, allowing them to incorporate at any point during pilus assembly and induce retraction. Our results suggest that the T4b pili, like the T4a pili and T2S pseudopili, are dynamic filaments that rapidly assemble and disassemble to perform their multiple and diverse functions, with each system having evolved distinct mechanisms to facilitate pilus dynamics. The *V*. *cholerae* minor pilin TcpB represents a simple but elegant multifunctional protein responsible for TCP dynamics and functions.

## Materials and Methods

### Bacterial strains and growth media

Bacterial strains used in this study are described in Table 1. All strains were maintained at -80°C in lysogeny broth (LB) containing 20% glycerol (v v^-1^). *V*. *cholerae* strains were grown under classical, TCP-expressing conditions of LB (starting pH of 6.5) at 30°C with aeration as previously described [7, 115]. Where appropriate, strains were grown with antibiotics at the following final concentrations: ampicillin (Ap) 100 μg/ml, gentamicin (Gm) 30 μg/ml, kanamycin (Km) 45 μg/ml, and streptomycin (Sm) 100 μg/ml, spectomycin (Sp), tetracyclin (Tet). All DNA manipulations were performed using standard molecular and genetic techniques [116, 117]. Expression of *tcpB* and *tcpB* mutants from p*tcpB* was induced using rhamnose at the indicated concentrations.

**Table 1:** List of bacterial strains, plasmids and primers

Reagent	Description or nucleotide sequence	Source / Reference
**Strains**		
*V*. *cholerae* O395	Classical O1, Ogawa, Sm	[7]
*V*. *cholerae* RT4031	Δ*tcpA*, major pilin deletion strain	[35]
*V*. *cholerae* RT4368	Δ*tcpB*, minor pilin deletion strain	[103]
*V*. *cholerae* RT4369	Δ*tcpC* secretin deletion strain	[103]
*V*. *cholerae* RT4634	TcpB_E5V_	[102]
*V*. *cholerae* TJK189	Flagella minus O395 Δ*flaA*	[118]
*V*. *cholerae* CF12	Δ*flaA*, Δ*tcpB*	This study
*V*. *cholerae* YG1257	Δ*flaA*, TcpB_E5V_	This study
*E*. *coli* DH5α	*F− endA1 glnV44 thi-1 recA1 relA1 gyrA96 deoR nupG Φ80dlacZΔM15 Δ(lacZYA-argF)U169*, *hsdR17(rK− mK+)*, *λ–*	Life Technologies
*E*. *coli* SHuffle^®^ T7 Express	*MiniF lysY (Cm*^*R*^*) / fhuA2 lacZ*::*T7 gene1 [lon] ompT ahpC gal λatt*::*pNEB3-r1-cDsbC* (*Sp*^*R*^, *lacI*^*q*^*)ΔtrxB sulA11 R(mcr-73*::*miniTn10—Tet*^*S*^*)2 [dcm] R(zgb-210*::*Tn10—Tet*^*S*^*) endA1 Δgor Δ(mcrC-mrr)114*::*IS10*	New England Biolabs
**Plasmids**		
pBAD22	pMB1, Para promoter, araC, Ap^R^	ATCC
pJMA10	pBAD22 derivative; *araC* replaced with P*rhaB*. *Bla*, Ap^R^	[102]
pJMA10.1	pJMA10 with *NcoI* site removed	This study
p*tcpB*	pJMA10.1 containing the *tcpB* gene	This study
p*tcpB*_E5D_	p*tcpB* Glu5Asp	This study
p*tcpB*_E5Q_	p*tcpB* Glu5Gln	This study
p*tcpB*_E5V_	p*tcpB* Glu5Val	This study
pET15b-*tcpA*	pET-15b expressing TcpA_29-199_, Ap^R^	[17]
pET15b-*tcpB*	pET-15b expressing TcpB_25-423_	This study
pTK20	pKAS32 Δ*flaA*; Ap^R^	[118]
pTK2	pKAS32 Δ*tcpB*; Ap^R^	[118]
pTRNS101	pKAS32 *tcpB*; Ap^R^	[102]
pCHG006	pKAS32 *tcpB*:E5V; Ap^R^	[102]
pKAS32	pGP704 *rpsL*; Ap^R^	[119]
**Primers**		
ABN26	GATCGGGCGCCTGAGCCTCGGTTGTGTGGTG	This study
ABN27	GATCGCCATGGTGAATTCCTCCTGAATTTCATTACGACCAGTC	This study
pJMA10-NcoDEL-F-NheI	TACTATCTTCAAAGCCACATTCGGTCGAAA	This study
pJMA10-NcoDel-R-KpnI	CGGGGTACCTTCCTCCTGAATTTCATTACGACCAGTCTAA	This study
VC-TcpB-F-KpnI	CGGGGTACCATGAGAAAATACCAACAAGGTGTCG	This study
VC-TcpB-R-XbaI	ATATATTCTAGATTAATTTTCACACCATTGAAACGCTATAAAA	This study
VC-tcpB(E5D)-F-KpnI	CGGGGTACCATGAGAAAATACCAACAAGGTGTC GGATTATTGGATGCG	This study
VC-TcpB(E5Q)F-KpnI	CGGGGTACCATGAGAAAATACCAACAAGGTGTCGGATTATTGCAGGCG	This study
VC-TcpB(E5V)F-KpnI	CGGGGTACCATGAGAAAATACCAACAAGGTGTCGGATTATTGGTGGCG	This study
TcpB-R-142-XbaI	ATATATTCTAGATTATTGTTTTATTTGTCGTTGAATTTCCG	This study
TcpB-R-217-XbaI	ATATATTCTAGATTACGAAAATTTTCTTTTAAAAGCGACGAAA	This study
TcpB-R-228-XbaI	ATATATTCTAGATTAAGCAAGACTGACCGCCAACG	This study
TcpB25-vc-fpcr-nde1	GGAATTCCATATGAAGCGGGAAGCTGAACTCATGATTAAA	This study
TcpB25-vc-rpcr-bamh1	CGCGGATCCTTAATTTTCACACCATTGAAACGCT	This study

### Plasmid and strain construction

Plasmids used in this study are listed in Table 1. Expression vector pJMA10 is derived from pBAD22 and constructed as follows. Primers ABN26/27 were used to amplify the rhamnose promoter P_RHA_ from *E*. *coli* BL21 chromosomal DNA. The PCR amplified region was then cloned into pBAD22-TOPO (ATCC), replacing the *araC* promoter region to generate pJMA10. This vector was further modified to remove the endogenous NcoI restriction digest sequence from the multiple cloning site (MCS) to prevent a possible frameshift mutation. Primers NcoDEL-F-NheI and NcoDEL-R-KpnI were used to PCR amplify a 1.7 kb fragment of pJMA10 upstream of the MCS that contains a unique NheI restriction site. The fragment downstream of the MCS was directly digested from the purified plasmid. Both fragments were digested with restriction enzymes NheI and KpnI, and ligated together using T4 DNA ligase (New England Biolabs) to generate the corrected plasmid pJMA10.1. Both pJMA10 and pJMA10.1 were verified by DNA sequencing.

The vector expressing the minor pilin TcpB was derived from expression vector pJMA10.1, which contains an Ap^R^ marker. The gene fragment encoding TcpB was PCR amplified with primers TcpB-F-KpnI/-R-XbaI from *V*. *cholerae* WT strain O395 genomic DNA. The PCR product was purified, digested with KpnI and XbaI, and ligated into pJMA10.1 at the KpnI/XbaI restriction sites using T4 DNA ligase. The construct p*tcpB* was verified by DNA sequencing and transformed into *V*. *cholerae* strains for complementation testing. Pilin expression was induced using rhamnose at indicated concentrations.

The conserved Glu5 residue in TcpB was changed to Asp, Gln and Val on p*tcpB* to generate p*tcpB*_E5D_, p*tcpB*_E5Q_ and p*tcpB*_E5V_ respectively. Forward primers TcpB-E5D/E5Q/E5V-F-KpnI were used individually with reverse primer TcpB-R-XbaI to PCR amplify from p*tcpB* the *tcpB* gene fragments encoding the corresponding amino acid substitutions E5D, E5Q and E5V. PCR products were purified and subcloned into pJMA10.1 as previously described. All plasmids were verified by DNA sequencing and transformed into *V*. *cholerae* Δ*tcpB* and cells were grown on LB-Sm/Ap plates.

### Generation of the *tcpB*-E5V chromosomal mutations

The Δ*tcpB*/*flaA* and *tcpB*-E5V/Δ*flaA* chromosomal mutants were generated by allelic exchange. pTRNS101 encoding *tcpB*-E5V [102] and pTK2 encoding *tcpB* with a central deletion [103] were used to introduce the E5V substitution and the Δ*tcpB* deletion into the *V*. *cholerae* Δ*flaA* strain TJK189 [118] by allelic exchange [119].

### Immunoblotting

Fractions of overnight cell cultures were analyzed by SDS-PAGE and immunoblotting to compare TcpA, TcpB and TcpF production in the various *V*. *cholerae* strains and conditions. Whole cell culture (WCC) fractions used to assess total protein were obtained by resuspending overnight cultures by vortexing. Sheared supernatant (SS) fractions were obtained by homogenizing the resuspended cells using an IKA ULTRA-TURRAX T8.01 disperser (IKA-Werke) at the maximum setting for 20 seconds [8]. Cell debris was removed by centrifugation at 13,000 x *g* for 30 minutes at RT using a bench top microfuge, with the supernatant representing the SS fraction. For analysis of secreted TcpF in the culture supernatant (CS), overnight cultures were centrifuged at 3000 x *g* for 10 minutes at RT to remove the cells and the culture supernatant containing secreted TcpF was filtered through a 0.22 μm syringe-drive filter (Pall) to remove remaining cells. Each fraction was mixed with Laemmli sample buffer (60 mM Tris pH 6.8, 5% 2-mercaptoethanol, 2% SDS, 10% glycerol, 0.02% bromophenol blue) and boiled for 10 minutes prior to loading 25 μl onto 15% SDS polyacrylamide gels. The PageRuler Unstained Protein Ladder (Fermentas) was included for mass markers. Proteins were transferred onto polyvinylidene difluoride (PVDF) membrane (Bio-Rad) at 4°C in transfer buffer (25 mM Tris, 192 mM glycine, 20% methanol) with a wet transfer apparatus (Bio-Rad). The membrane was blocked for 1 hour at RT with non-fat dried milk (5% w/v) in Tris-buffered saline with 0.1% Tween. Proteins were detected with rabbit polyclonal antisera raised against TcpA peptide 174–199 [120], TcpB peptide 64–78 (Pacific Immunology) and mouse monoclonal antisera for TcpF [10]. Goat-anti-rabbit or goat-anti-mouse secondary antibodies conjugated to horseradish peroxidase (Jackson ImmunoResearch) were used to bind the primary antibody. The protein mass markers were detected with Strep-Tactin-HRP conjugate (IBA). Immunoblots were visualized by enhanced chemiluminescence using the SuperSignal West Pico substrate (Thermo Fisher Scientific). Immunoblots were digitized using the FujiFilm LAS 4000 imager.

### Expression and purification of ΔN-TcpA and ΔN-TcpB

Expression and purification of ΔN-TcpA displaying an N-terminal hexahistidine tag (His) and linker are described elsewhere [17]. The gene fragment encoding ΔN-TcpB (residues 25–423) was PCR amplified with primers TcpB25-vc-fpcr-nde1 and TcpB25-vc-rpcr-BamH1 from *V*. *cholerae* WT strain O395 genomic DNA, followed by digestion with restriction enzymes NdeI and BamHI. The product was ligated into expression vector pET15b (Novagen), which encodes a His-tag and linker with a thrombin cleavage site. The construct was transformed into SHuffle T7 Express *lysY* Competent *E*. *coli* (New England Biolabs) by heat shock. The cells were grown in LB broth supplemented with ampicillin (100 μg/ml) to OD_600_ of 0.1 in a shaking incubator 37°C, and protein expression was induced by adding isopropyl β-D-1-thiogalactopyranoside (IPTG, final concentration to 0.1 mM). The temperature was reduced to 14°C and cells were grown for a further 20 hours and harvested by centrifugation (5000 x *g*, 30 min, 4°C). The culture supernatant was discarded and the cell pellet was resuspended in lysis buffer (50 mM Na_2_HPO_4_/NaH_2_PO_4_ pH 7.4, 20 mM Tris HCl pH 7.4, 100 mM NaCl, EDTA-free protease inhibitor cocktail [Roche], and 1 mg/ml lysozyme) and gently stirred at room temperature for an hour. Cells were lysed by sonication and cell debris was removed by centrifugation at 40,000 x g for 1 hour at 4°C.

Purification was carried out at 4°C. The cell lysate was filtered through 0.4 μm polyethersulfone membrane and loaded onto a Ni-NTA column pre-equilibrated with wash buffer (50 mM Na_2_HPO_4_/NaH_2_PO_4_ pH 7.4, 30 mM imidazole and 100 mM NaCl). After washing the column with 10 column volumes of wash buffer, the N-terminal His tag was cleaved off of the TcpB bound to the Ni-NTA column by thrombin digestion and the protein was eluted with elution buffer (50 mM Na_2_HPO_4_/NaH_2_PO_4_ pH 7.4, 250 mM imidazole, 100 mM NaCl). The protein was further purified by size exclusion chromatography using a HiPrep 26/60 Sephacryl S-100 HR column (GE Healthcare) in buffer containing 20 mM Tris-HCl, pH 7.4, 100 mM NaCl, 0.5 mM EDTA, 0.5 mM EGTA and concentrated to 10 mg/ml using a stirred-cell concentrator (Millipore). The purity was estimated to be >95%.

### Purification of TCP^H181A^

*V*. *cholerae* strain RT4225 expressing a mutation in *tcpA* encoding a His181Ala substitution [35], was grown on a LB plate containing ampicillin (LB/Ap). A single colony was used to inoculate 1 ml of LB/Ap, which was incubated at 37°C on a rotary shaker for 30 min. The cell culture was diluted to OD_600_ = 0.01 and 400 μl was used to inoculate 200 ml of LB, pH 6.5, 0.4 mM IPTG, 100 μg/ml Ap in a 2 L-flask, which was incubated at 30°C for 18 hours shaking at 250 rpm. Four ml of 0.5 M EDTA (pH 8) and 2 ml of 0.1 M histidine-HCl were added to the culture, which was centrifuged at 5000 x*g* for 15 minutes at 4°C to pellet cells. The supernatant was centrifuged again at 5000 x*g* for 10 minutes at 4°C to pellet residual cells. After transferring the supernatant to new tubes, solid ammonium sulfate (AmSO_4_) was added to 10% saturation and the solution was incubated at 4°C for 2 hours on a rocker. The solution was centrifuged at 10,000 x*g* for 30 minutes at 4°C, and AmSO_4_ was added to 30% saturation. The solution was incubated at 4°C for one hour on the rocker then centrifuged at 10,000 x*g* for 30 min at 4°C to pellet the pili. The pellet was resuspended in 200 μl of PBS containing 10 mM EDTA and dialyzed exhaustively using a 3500 kDa molecular weight cut-off membrane against precooled PBS, 10 mM EDTA.

### Autoagglutination assay

Cells were grown in 2 mL LB with antibiotics (streptomycin, ampicillin as required) for 2 hours at 37°C with rotation and cell concentrations were normalized to OD_600_ of 0.01. Normalized cultures were diluted 1/500 in 3 mL LB (pH 6.5) and grown overnight on a Ferris wheel rotator at 30°C with antibiotics and rhamnose as required. Overnight cultures were allowed to settle at room temperature for 15 minutes, after which the autoagglutination phenotype was assessed visually and the OD_600_ of the culture supernatant was measured. Experiments were performed in triplicate.

### Quantification of TcpA and TcpB in TCP^H181A^

ΔN-TcpA and ΔN-TcpB concentrations were determined by UV absorbance (260 nm) using a Nanodrop spectrophotometer (Thermo Fisher Scientific). Known amounts of each protein (standards) and fixed volumes of purified TCP^H181A^ were separated on a 15% SDS polyacrylamide gel. Proteins were transferred onto PVDF membrane and immunoblotted using anti-TcpA and anti-TcpB antibodies. Protein bands were scanned with a FujiFilm LAS4000 imager and analyzed using ImageJ [121]. Band densities of the ΔN-TcpA and ΔN-TcpB standards were normalized to the densest standard band in that blot to make averaging possible. The averages of relative densities from two replicates were plotted against their protein amounts and the plot was used to interpolate the amount of native TcpA or TcpB present in the purified TCP^H181A^ sample.

### Negative staining and immunogold TEM imaging

*V*. *cholerae* cells were grown under TCP-expressing conditions. Carbon-coated grids (CF-300, Electron Microscopy Science) were inverted on top of a 20 μL drop of sample on Parafilm and incubated for 10 minutes. Following incubation, grids were transferred to drops of Tris-buffered saline with 0.1% Tween (TBST). Grids were then stained with 3% uranyl acetate and imaged on a Hitachi 8100 STEM operating at 120 kEv.

For immunogold labeled samples, grids were inverted on top of a 25 μl drop of overnight cell culture on Parafilm in a humidified chamber to prevent evaporation and incubated at 30°C for 10 min. Following incubation, grids were transferred to drops of fixative (4% paraformaldehyde/0.2% glutaraldehyde in 0.2 M sodium cacodylate pH 7.4) for 1 hour. The grids were washed with Tris-buffered saline with 0.1% Tween (TBST) and blocked for 1 hour in bovine serine albumin (BSA) in TBST. Samples were incubated with primary rabbit antibody raised against TcpA (peptide 174–199 [120], 1:50 dilution in TBST 1% BSA), washed in TBST, and then incubated for 30 minutes with gold-conjugated anti-rabbit secondary antibody (1:60 dilution in TBST 1% BSA) (Jackson Immunoresearch, Electron Microscopy Sciences). The final wash in TBST was followed by staining with 3% uranyl acetate. Samples were imaged on a Hitachi 8100 STEM at 200 kV with the exception of Fig 2B, which was imaged on a JEOL 100CX TEM at 100 kV.

### Micropillars assays

Micropillars assays were performed as previously reported [107] using PoMPs (Polyacrylamide MicroPillars). Briefly, microfabricated silica molds were inverted onto 15 μL of a mixture of 20% polyacrylamide and 0.2% Bis-acrylamide with 1/10 volume of ammonium persulfate and 1/1000 volume of TEMED on activated 25 mm diameter round coverslips (Warner Instruments). Reticulation of the hydrogel and removal of the silica mold yielded a hexagonal array of polymerized micropillars spaced 3 μm center to center with a spring constant (*k*) of 25 +/- 4 pN/μm. The micropillars hydrogel was activated with sulfo-SANPAH crosslinker (Invitrogen) according to the manufacturer's recommendations, then the micropillars were treated with poly-L-lysine (30 μg/ml in phosphate buffered saline) for one hour at 37°C. After 3 washes in water the micropillars were coated for one hour with a 1/10 (V/V) solution of 20 nm carboxylated beads in water (Molecular Probes). After two washes with water and one with phosphate buffered saline, the coverslips supporting the micropillars were mounted at the bottom of an observation chamber (Attofluor chamber, Invitrogen). Two mls LB (pH 8.5) and 5 μL suspension of *V*. *cholerae* overnight culture was added to the chamber and incubated at 30°C for one hour. Ten Hz movies of the pillars tips were recorded using a 60X objective on an inverted microscope with an environmental chamber at 30°C (TiEclipse, Nikon). Positions of the micropillar tips were analyzed in the movies using a custom-designed cross-correlation algorithm [122]. The position of a non-deflected micropillar away from any colony was used as a reference to compensate for any global movement of the chamber. The forces exerted on the micropillars were calculated based of the displacement of the pillars and their calibrated spring constant using a commercial software package Matlab (Mathworks Inc. Natick, MA) and Igor Pro (WaveMetrics, Inc., Lake Oswego, OR).

The *V. cholerae* suspensions used to perform the micropillar assays were prepared as follows: Δ*flaA*, Δ*tcpB*/Δ*flaA* and *tcpB*-E5V/Δ*flaA* strains were streaked onto LB agar plates and grown for 24 hours at 37°C, a single colony was touched with a 2 mm sterilized loop and the bacteria attached to the loop were transferred to 1 ml of LB (pH 6.5). Ten μl of this solution was used to inoculate 3 ml LB (pH 6.5) in a culture tube, which was rotated at 30°C for 12 to 15 hours. For *V*. *cholerae* strain Δ*tcpB*/Δ*flaA*, 1 μl of anti-lipopolysaccharide antibody was added to induce bacterial aggregation and the suspension was rotated an additional 30 minutes prior to the micropillar assay.

## Supporting Information

S1 Fig Quantification of TcpA and TcpB in purified pili from the *V*. *cholerae tcpA*(H181A) mutant. **(A)** Top panel: Immunoblot of known amounts of recombinant N-terminally truncated TcpA (ΔN-TcpA) and purified TCP^H181A^ from *V*. *cholerae* RT4225 probed with anti-TcpA antibody. Bottom panel: graph of the ΔN-TcpA average band densities, determined by ImageJ analysis [121] from two replicates, plotted against the known protein amounts. From this graph the concentration of TcpA in purified TCP^H181A^ was found to be 12±1 mg/ml (SEM, n = 3) or 0.59 mM. The error bars are standard deviations. **(B)** Top panel: Immunoblot of known amounts of recombinant N-terminally truncated TcpB (ΔN-TcpB) and purified TCP^H181^ probed with anti-TcpB antibody. Bottom panel: graph of the ΔN-TcpB average band densities, determined by ImageJ analysis [121] from three replicates, plotted against the known protein amounts. From this graph the TcpB concentration in purified TCP^H181A^ was found to be 4±1 μg/ml (SEM, n = 4) or 86 nM. Thus, the stoichiometric ratio of TcpA:TcpB in TCP^H181A^ is ~ 7000:1. **(C)** TEM image of purified TCP^H181A^ and their dimensions.
(TIF)

S2 Fig Titration to determine the optimal rhamnose concentration for induction of TcpB expression. TcpB expression and autoagglutination levels were assayed in the *V*. *cholerae* Δ*tcpB* strain complemented with p*tcpB* and induced with varying rhamnose concentrations (shown as %, w/v). **(A)** Immunoblot of *V*. *cholerae* whole cell cultures (WCC) probed with anti-TcpB antibodies. The loading control is an unknown ~60 kDa protein present in the WCC fraction and detected by the Strep-Tactin-HRP conjugate. **(B)** Autoagglutination levels. The more complete the autoagglutination the lower the OD_600_ value. Values are averaged for 3 experiments; error bars represent standard deviations. Autoagglutination is closest to that of WT *V*. *cholerae* O395 with 0.001% rhamnose.
(TIF)

S3 Fig The non-motile *tcpB*-E5V/Δ*motAB* double mutant autoagglutinates. The *tcpB*-E5V/Δ*motAB* double mutant produces flagella and autoagglutinates at levels approaching WT, supporting the idea that the gain of autoagglutination observed for the *tcpB*-E5V/Δ*flaA* double mutant, as compared to that of the poorly autoagglutinating *tcpB*-E5V single mutant, is due to loss of motility rather than loss of flagella.
(TIF)

S1 Movie Micropillars assay of *V*. *cholerae* O395 Δ*flaA*. Differential contrast movie of the micropillars assay of *V*. *cholerae ΔflaA*. The average distance between pillars is 3 μm center to center and the full length of the movie is 4 min.
(ZIP)

S2 Movie Micropillars assay of *V*. *cholerae* O395 Δ*flaA*. Differential contrast movie of the micropillars assay of *V*. *cholera ΔflaA*. The average distance between pillars is 3 μm center to center and the full length of the movie is 6.5 min.
(ZIP)

S3 Movie Micropillars assay of *V*. *cholerae* Δ*tcpB*/Δ*flaA*. Differential contrast movie of the micropillars assay of *V*. *cholerae* Δ*tcpB/ΔflaA*. The average distance between pillars is 3 μm center to center and the full length of the movie is 4 min.
(ZIP)

S4 Movie Micropillars assay of *V*. *cholerae tcpB*-E5V/Δ*flaA*. Differential contrast movie of the micropillars assay of *V*. *cholerae tcpB*-E5V/Δ*flaA*. The average distance between pillars is 3 μm center to center and the full length of the movie is 6.5 min.
(ZIP)
