# Supplementary figures and images for: The Vibrio cholerae Minor Pilin TcpB Initiates Assembly and Retraction of the Toxin-Coregulated Pilus
Source: PLoS Pathog. 2016 Dec 19;12(12):e1006109. doi: 10.1371/journal.ppat.1006109 (PMC5207764; doi:10.1371/journal.ppat.1006109)

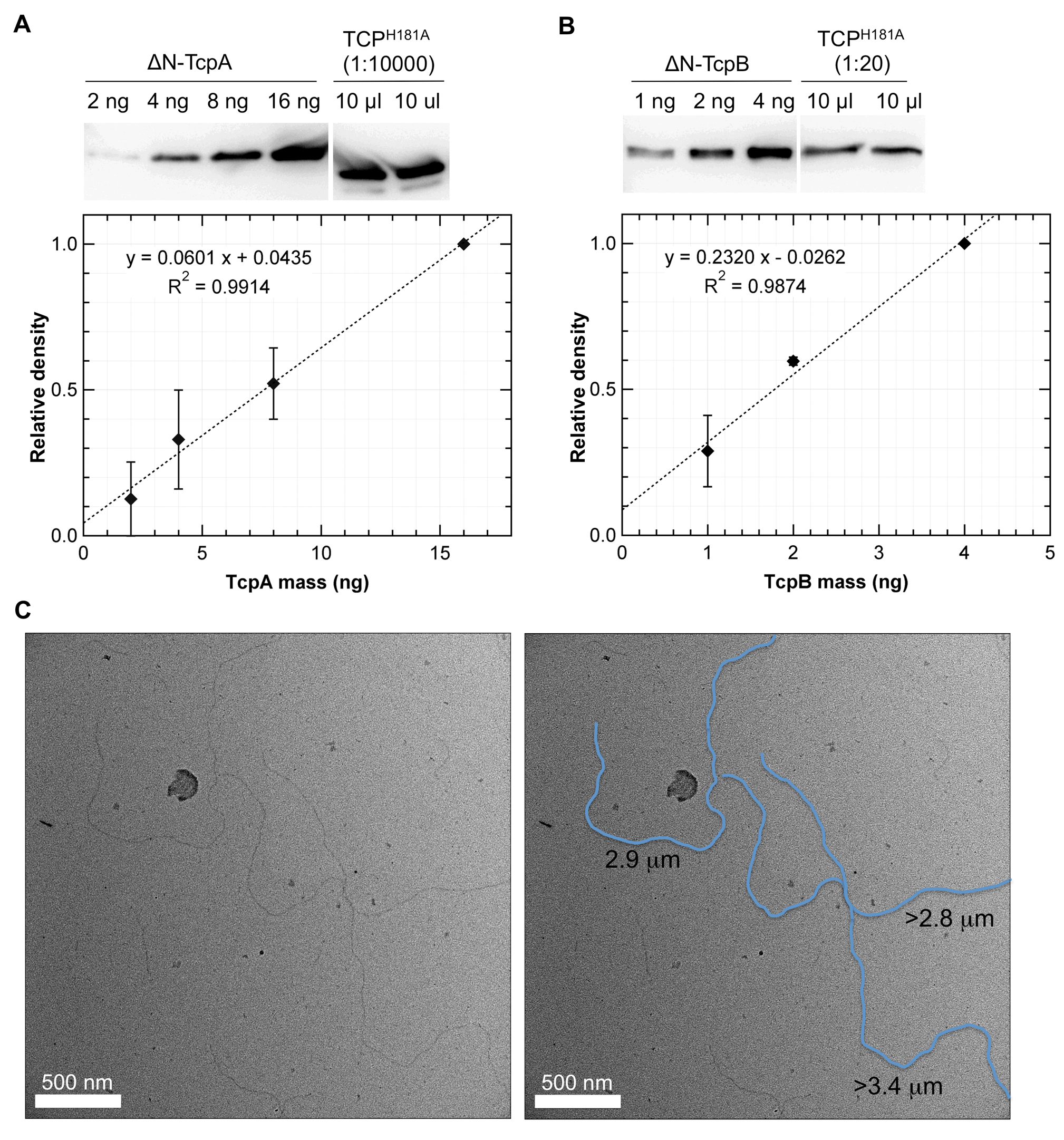

Supplement: S1 Fig — (A) Top panel: Immunoblot of known amounts of recombinant N-terminally truncated TcpA (ΔN-TcpA) and purified TCPH181A from V. cholerae RT4225 probed with anti-TcpA antibody. Bottom panel: graph of the ΔN-TcpA average band densities, determined by ImageJ analysis [121] from two replicates, plotted against the known protein amounts. From this graph the concentration of TcpA in purified TCPH181A was found to be 12±1 mg/ml (SEM, n = 3) or 0.59 mM. The error bars are standard deviations. (B) Top panel: Immunoblot of known amounts of recombinant N-terminally truncated TcpB (ΔN-TcpB) and purified TCPH181 probed with anti-TcpB antibody. Bottom panel: graph of the ΔN-TcpB average band densities, determined by ImageJ analysis [121] from three replicates, plotted against the known protein amounts. From this graph the TcpB concentration in purified TCPH181A was found to be 4±1 μg/ml (SEM, n = 4) or 86 nM. Thus, the stoichiometric ratio of TcpA:TcpB in TCPH181A is ~ 7000:1. (C) TEM image of purified TCPH181A and their dimensions. (TIF) [file ppat.1006109.s001.tif]

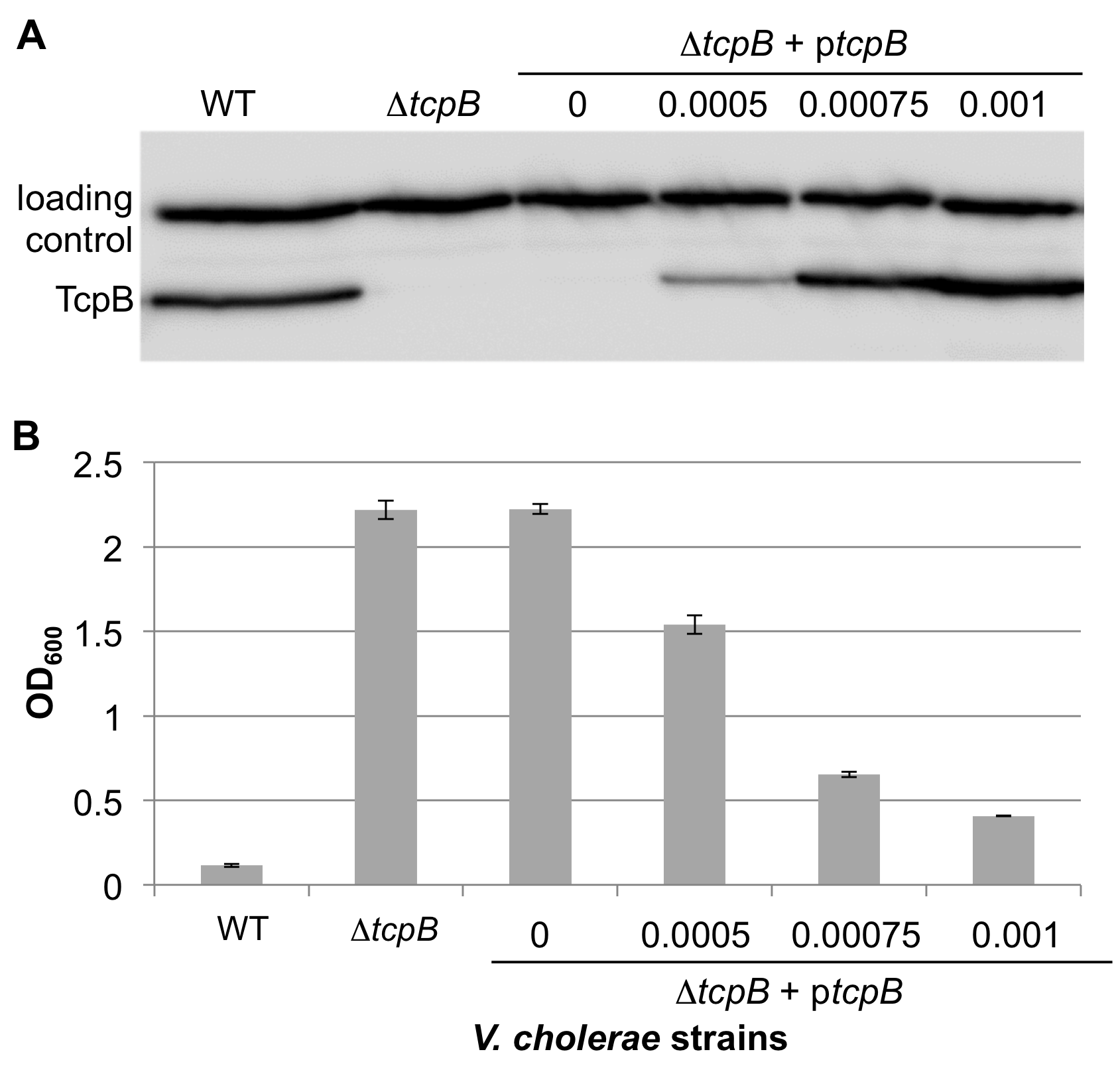

Supplement: S2 Fig — TcpB expression and autoagglutination levels were assayed in the V. cholerae ΔtcpB strain complemented with ptcpB and induced with varying rhamnose concentrations (shown as %, w/v). (A) Immunoblot of V. cholerae whole cell cultures (WCC) probed with anti-TcpB antibodies. The loading control is an unknown ~60 kDa protein present in the WCC fraction and detected by the Strep-Tactin-HRP conjugate. (B) Autoagglutination levels. The more complete the autoagglutination the lower the OD600 value. Values are averaged for 3 experiments; error bars represent standard deviations. Autoagglutination is closest to that of WT V. cholerae O395 with 0.001% rhamnose. (TIF) [file ppat.1006109.s002.tif]

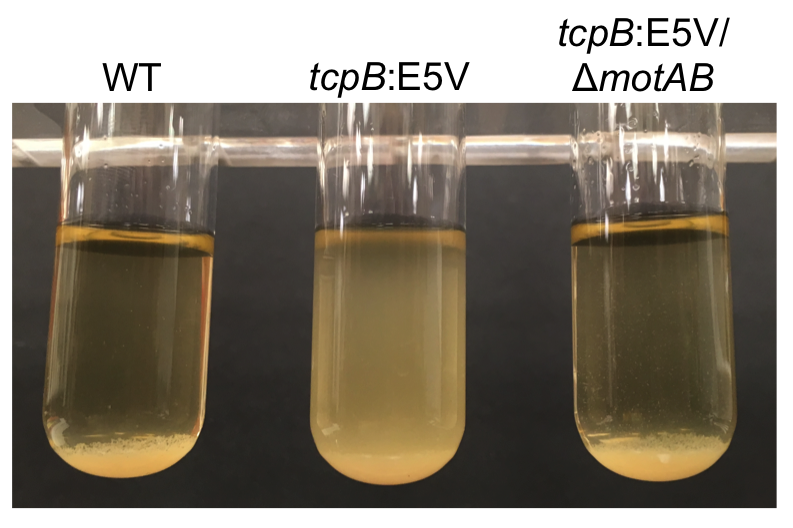

Supplement: S3 Fig — The tcpB-E5V/ΔmotAB double mutant produces flagella and autoagglutinates at levels approaching WT, supporting the idea that the gain of autoagglutination observed for the tcpB-E5V/ΔflaA double mutant, as compared to that of the poorly autoagglutinating tcpB-E5V single mutant, is due to loss of motility rather than loss of flagella. (TIF) [file ppat.1006109.s003.tif]
